# Rh-doped MoSe_2_ as a toxic gas scavenger: a first-principles study

**DOI:** 10.1039/c8na00233a

**Published:** 2018-11-09

**Authors:** Hao Cui, Guozhi Zhang, Xiaoxing Zhang, Ju Tang

**Affiliations:** State Key Laboratory of Power Transmission Equipment & System Security and New Technology, Chongqing University Chongqing 400044 China xiaoxing.zhang@outlook.com; School of Electrical and Computer Engineering, Georgia Institute of Technology Atlanta 30332 GA USA; School of Electrical Engineering, Wuhan University Wuhan 430072 China

## Abstract

Using first-principles theory, we investigated the most stable configuration for the Rh dopant on a MoSe_2_ monolayer, and the interaction of the Rh-doped MoSe_2_ (Rh-MoSe_2_) monolayer with four toxic gases (CO, NO, NO_2_ and SO_2_) to exploit the potential application of the Rh-MoS_2_ monolayer as a gas sensor or adsorbent. Based on adsorption behavior comparison with other 2D adsorbents and desorption behavior analysis, we assume that the Rh-MoSe_2_ monolayer is a desirable adsorbent for CO, NO and NO_2_ storage or removal given the larger adsorption energy (*E*_ad_) of −2.00, −2.56 and −1.88 eV, respectively, compared with other materials. In the meanwhile, the Rh-MoSe_2_ monolayer is a good sensing material for SO_2_ detection according to its desirable adsorption and desorption behaviors towards the target molecule. Our theoretical calculation would provide a first insight into the TM-doping effect on the structural and electronic properties of the MoSe_2_ monolayer, and shed light on the application of Rh-MoSe_2_ for the sensing or disposal of common toxic gases.

## Abstract 2

Using first-principles theory, we investigated the most stable configuration for the Rh dopant on a MoSe_2_ monolayer, and the interaction of the Rh-doped MoSe_2_ (Rh-MoSe_2_) monolayer with four toxic gases (CO, NO, NO_2_ and SO_2_) to exploit the potential application of the Rh-MoS_2_ monolayer as a gas sensor or adsorbent.

## Graphical abstract

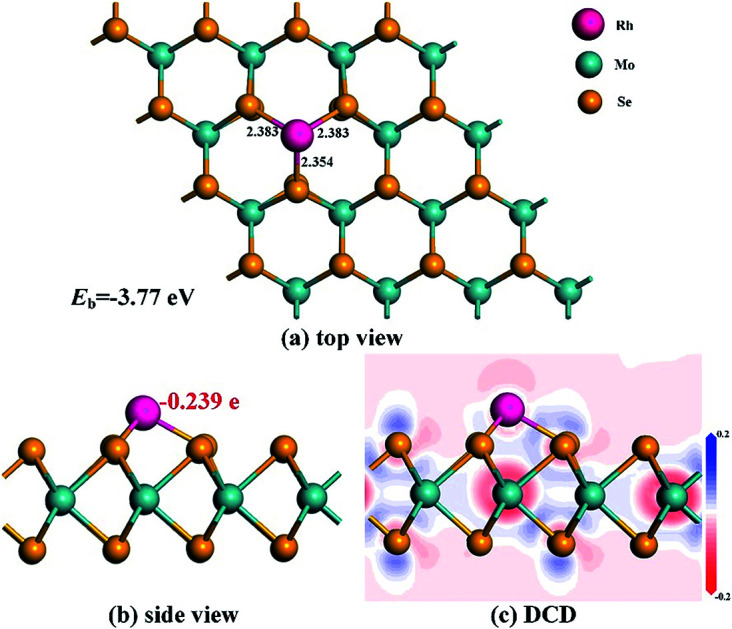

## 1. Introduction

Sensing toxic gases, especially detection of the ones at sub-ppm levels, is quite imperative with respect to environmental standards and agricultural pollution monitoring. In this regard, researchers have always contributed to finding novel sensing materials for potential application as chemical sensors with a rapid response, high sensitivity and low cost. In the past few decades, semiconducting metal oxide nanowire,^1–3^ carbon nanotube,^4–6^ and graphene^7–9^ based sensors have successively caught the attention of researchers, arousing considerable interest from the research community. Nevertheless, they are never satisfied by these achievements, persisting to pursue some new materials that possess more fascinating sensing behavior than the previous ones.

After the successful application of graphene as a gas sensor, scholars have turned their attention to two-dimensional (2D) materials that have large surface–volume ratios and tunable electronic properties due to their unique structural configuration. Materials such as hexagonal boron nitride,^10^ antimonene,^11^ phosphorene^12^ and silicene^13^ have become the focus of the sensing field, in order to find candidate materials having the advantages of graphene such as high carrier mobility and strong chemical activity for gas interaction,^14,15^ as well as semiconducting properties. In the meanwhile, group III–V nitrides, particularly AlN and InN,^16,17^ have been regarded as promising structures for gas sensing,^18,19^ and the experimental breakthrough in the synthesis of InN^20^ makes it possible to be used as a substitute for graphene with inherent bandgap characteristics.^21^

Very recently, 2D transition metal dichalcogenides (TMDs), especially MoS_2_ monolayers,^22–24^ have attracted much attention as alternative materials to conventional metal oxides for chemical sensing devices. Moreover, surface doping with transition metal (TM) atoms has been demonstrated to provide a monolayer with enhanced adsorption and sensing performance for gas molecules^25–28^ due to the improved chemical activity and electron mobility induced by the TM dopant,^29^ opening up a novel insight into the sensor family. Other than that, MoSe_2_, as a new emerging semiconducting material, has been investigated as well for its application as a sensor. While Late *et al.* first reported the high sensing performance of the MoSe_2_ monolayer for ppm-level NH_3_ gas,^30^ Baek *et al.* developed a MoSe_2_ multilayer based field-effect transistor for NO_2_ detection.^31^ However, the potential applications of MoSe_2_ based materials need to be further explored and broadened after their successful synthesis by chemical vapor deposition.^32^ This inspires us to implement a first-principles calculation to study the adsorption performance of TM-MoSe_2_ monolayers towards four industrial exhaust gases including CO, NO, NO_2_ and SO_2_ to put forward a novel material for toxic gas sensing. Among numerous TM atoms, rhodium (Rh) is the one with strong electron mobility and catalytic performance for gas interaction, previously demonstrated by carbon nanotube,^33^ graphene,^34^ and MoS_2_ monolayer systems^35^ where Rh was proposed as a dopant to functionalize the proposed surfaces. We assumed that it would be interesting and necessary to investigate the adsorption and sensing behaviors of the Rh-doped MoSe_2_ (Rh-MoSe_2_) monolayer towards toxic gases to exploit the novel material for toxic gas detection or removal. The results indicate that the Rh-MoSe_2_ monolayer possesses quite strong adsorption behavior towards CO, NO and NO_2_ molecules that gives rise to chemisorption in these systems, while physisorption could be determined for the Rh-MoSe_2_/SO_2_ system. Through adsorption behavior comparison with other 2D adsorbents and desorption behavior analysis, we assume that Rh-MoSe_2_ is a desirable adsorbent for CO, NO and NO_2_ storage or removal while being a good sensing material for SO_2_ detection. To the best of our knowledge, this would be the first report investigating the potential application of Rh-MoSe_2_ for the removal of four toxic species in a theoretical manner.

## 2. Computational details

In this work, spin-polarized calculations were implemented in the Dmol^3^ package^36^ of Materials Studio. The Perdew–Burke–Ernzerhof (PBE) functional with a generalized gradient approximation (GGA) was employed to deal with the electron exchange and correlation,^37^ and to obtain the optimized structures. The Grimme method was employed^38^ for better understanding the van der Waals interaction. We selected double numerical plus polarization (DNP) as the atomic orbital basis set,^26^ while the DFT semi-core pseudopotential (DSSP) method was employed to dissolve the relativistic effect of the TM atom.^39^ The *k*-point sample of the Monkhorst–Pack grid was sampled to 5 × 5 × 1 of the Brillouin zone for geometry optimization and to 7 × 7 × 1 for electronic structure calculations.^40^ The energy tolerance accuracy, maximum force, and displacement were selected as 10^−5^ Ha, 2 × 10^−3^ Ha Å^−1^, and 5 × 10^−3^ Å,^41^ respectively. For static electronic structure calculations, a self-consistent loop energy of 10^−6^ Ha, global orbital cut-off radius of 5.0 Å and smearing of 0.005 Ha were employed to ensure the accurate results of total energy.^42^ For basis set superposition errors (BSSE), little impact could be caused in the Dmol^3^ package,^43^ and thus we would not analyze it in the following part.

A 4 × 4 × 1 MoSe_2_ monolayer supercell including 16 Mo and 32 Se atoms with a vacuum region of 15 Å was established and relaxed to its most stable configuration. A previous report has proved that a 4 × 4 supercell would be large enough to conduct the gas adsorption process while a 15 Å slab would be appropriate to prevent the interaction between adjacent units.^44^ The lattice constant calculated here was 3.30 Å, which is in agreement with other theoretical studies (3.31 Å (ref. 45)).

The adsorption energy (*E*_ad_) of each gas adsorption process was calculated by the following equation:^23^

1 *E*_ad_ = *E*_Rh-MoSe_2_/gas_ − *E*_Rh-MoSe_2__ − *E*_gas_

where the *E*_Rh-MoSe_2_/gas_, *E*_Rh-MoSe_2__ and *E*_gas_ represent energies of the adsorbed system, isolated Rh-MoSe_2_ and gas molecule, respectively. To analyze the charge transfer (*Q*_T_) between the target molecule and adsorbent surface, the Mulliken population analysis was considered, characterized by the carried electron value by gas molecules after adsorption. Only the most favorable configuration for gas adsorption would be plotted and analyzed in the following parts.

## 3. Results and discussion

### 3.1 Geometric and electronic structure of Rh-MoSe_2_

We determined Rh atom adsorption onto a bare MoSe_2_ monolayer Rh-MoSe_2_, where four possible sites were considered, traced as T_H_ (above the center of the hexagonal ring of MoSe_2_), T_Mo_ (at the top of the Mo atom), T_Se_ (at the top of the Se atom) and B_S–S_ (the bridge site between two Se atoms), respectively. The binding energy (*E*_b_) for Rh adsorption onto the most favorable doping site is determined through the formula:

2 *E*_d_ = *E*_Rh-MoSe_2__ − *E*_Rh_ − *E*_MoSe2_

where *E*_Rh-MoSe_2__, *E*_Rh_, and *E*_MoSe_2__ represent the energies of the Rh-MoSe_2_, Rh atom and pure MoSe_2_, respectively.

**Fig. 1:**
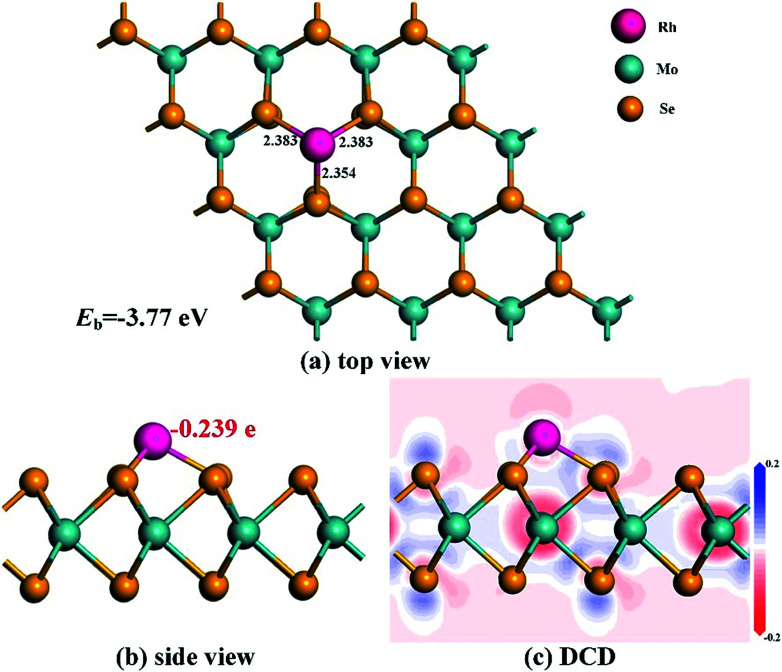
Geometric structures (a) and (b) and DCD (c) of the Rh-MoSe_2_ monolayer. The black values are the atom-to-atom distance while the red value is the electron amount carried by Rh dopant.

After optimization, the most stable configuration of Rh-MoSe_2_ in line with relevant deformation charge density (DCD) is shown in Fig. 1. One can see that the Rh atom tends to be adsorbed onto the MoSe_2_ monolayer through the T_Mo_ site, with three Rh–Se bond lengths of 2.383, 2.383 and 2.354 Å, respectively. The geometry of MoSe_2_ undergoes a little deformation after Rh doping due to the binding force of Rh–Se bonds.^46^ The *Q*_T_ between the Rh dopant and the MoSe_2_ monolayer is found to be −0.239 e, indicating that the Rh dopant acts as an electron acceptor while the MoSe_2_ monolayer acts as a donor. This is in line with the strong electron withdrawing properties of the Rh atom,^33^ thereby leading to high electron localization around the Rh atom. In addition, we can find that the Mo atoms are mainly the electron accepting centers while the Se atoms are the electron donating centers for the optimized Rh-MoSe_2_ system.

**Fig. 2:**
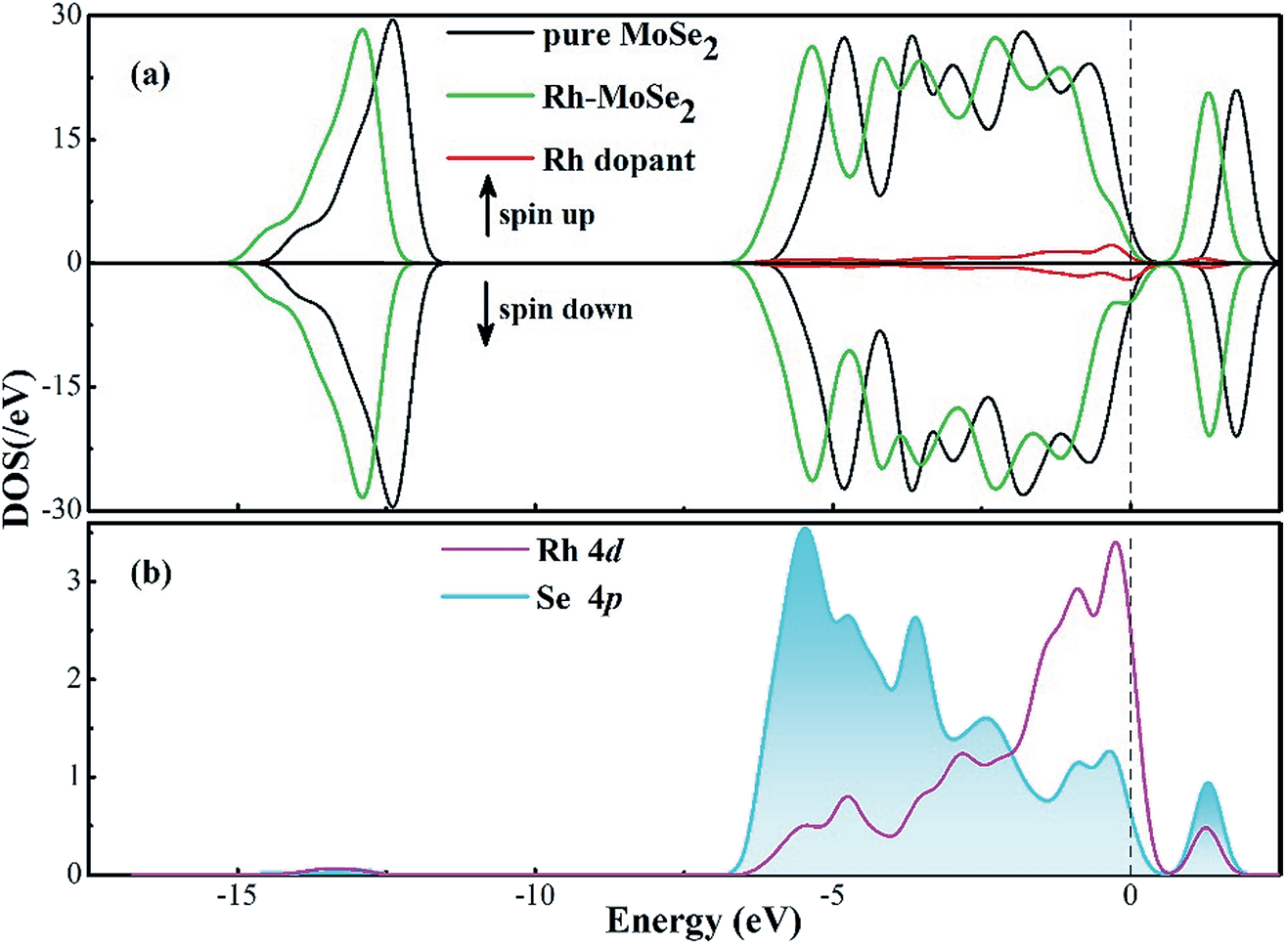
DOS distribution of Rh-MoSe_2_. The dashed line is the Fermi level.

To further elucidate the electronic behavior of the Rh doping effect on the MoSe_2_ monolayer compared with its pure counterpart, density of states (DOS) analysis is performed, as depicted in Fig. 2. It can be seen in the total DOS distribution of Fig. 2(a) that there is a big band gap near the Fermi level in the DOS curve of intrinsic MoSe_2_, confirming its semiconducting properties well. With the doping of the Rh atom, the gap gets narrowed obviously due to the upward shifted Fermi level which was originally identified as the valence band maximum in the Dmol^3^ package, caused by the Rh contribution. It is interesting to note that the DOS spin up and down of the Rh-MoSe_2_ monolayer are shown to be asymmetric in comparison with those in the pure MoSe_2_ system that shows good symmetry, which could be ascribed to the total magnetic moment of 1.0 *μ*_B_ in this system induced by the Rh dopant. Moreover, due to the electron-donating behavior of the MoSe_2_ monolayer that results in an improved effective coulombic potential,^47^ the DOS curve of the Rh-MoSe_2_ system is found to left shift towards a lower region consequently compared with that of its intrinsic counterpart. In Fig. 1(b), the DOS of the Rh 4d orbital is largely overlapped with that of the Se 4p orbital, demonstrating strong hybridization of the Rh atom onto the MoSe_2_ monolayer. Apart from that, it reveals that the highest occupied molecular orbitals (HOMOs) are mainly located at the Se atom while the lowest unoccupied molecular orbitals are at the Rh atom, which confirms the results of DCD that charge is accumulated around the Rh dopant.^48^

### 3.2 CO adsorption

**Fig. 3:**
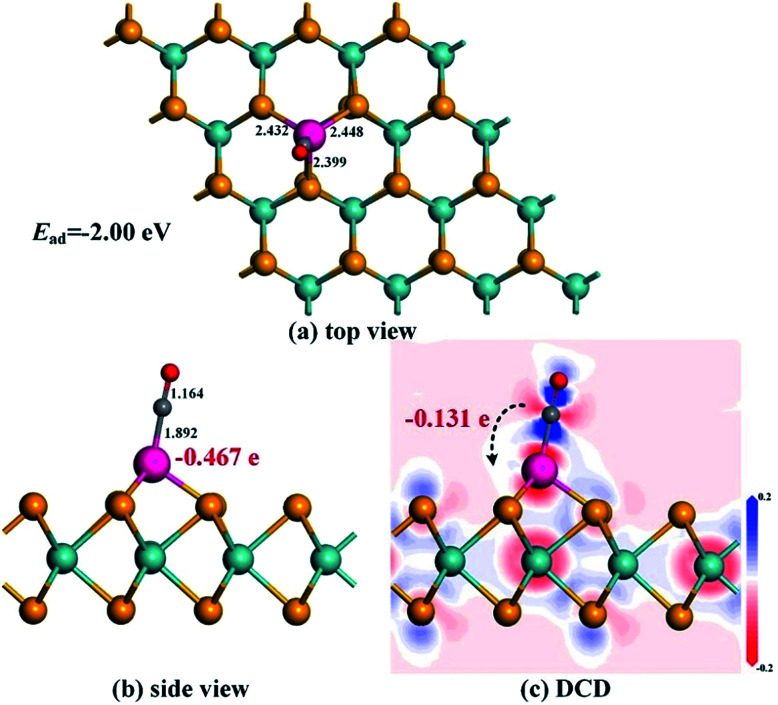
Adsorption configuration (a) and (b) and DCD (c) of the Rh-MoSe_2_/CO system.

Fig. 3 shows the most stable configuration and related DCD for CO adsorption on the Rh-MoSe_2_ monolayer. One can see that the C atom is trapped by the Rh dopant with a Rh–C bond length of 1.892 Å after the CO molecule is adsorbed on the surface. It would be worth noting that such a value of atom-to-atom distance is even shorter than the sum of the corresponding covalent radii (2.00 Å for Rh–C^49^) indicating some chemisorption in this system.^50^ In fact, the large enough *E*_ad_ of −2.00 eV here could not only confirm the strong adsorption performance of Rh-MoSe_2_ towards the CO molecule, but also indicate its chemical nature with strong spontaneity for this interaction. In the meanwhile, three Rh–Se bonds, after CO adsorption, are elongated to 2.432, 2.448 and 2.399 Å, respectively, and the C–O bond of the CO molecule is prolonged to 1.164 Å from 1.142 Å in an isolated molecule. These findings suggest the activation behavior for the CO molecule when interacted with the monolayer. The DCD shows that the Rh-MoSe_2_ monolayer acting as an electron acceptor withdraws 0.131 e from the adsorbed CO molecule. This, combined with the Mulliken population analysis for the Rh dopant that carries 0.467 e after adsorption, indicates that the Rh dopant is an electron localization center accepting charge from both gas molecules and the MoSe_2_ surface.

**Fig. 4:**
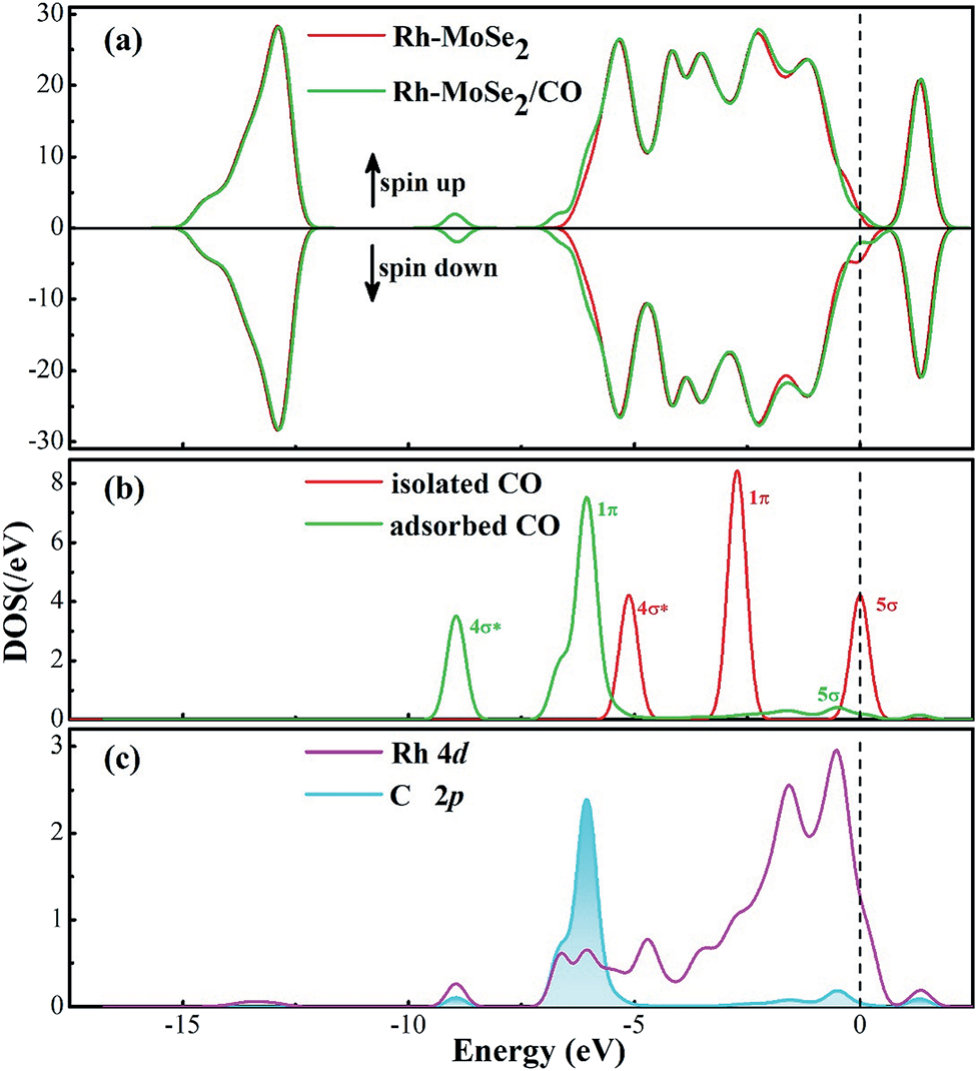
DOS distribution of the Rh-MoSe_2_/CO system. The dashed line is the Fermi level. In the gas DOS, the red value shows the orbitals of isolated molecules while the green one shows the orbitals of adsorbed molecules.

Fig. 4 shows the total and partial DOS distributions for the CO adsorption system. Based on the comparison between the total DOS for the pure Rh-MoSe_2_ system and the one for the adsorbed system, we can find that they are basically overlapped except for the area near the Fermi levels where the Rh dopant contributes a lot due to its strong electron activity, and the areas at 6.5 and 8.5 eV where the adsorbed CO molecule makes a great contribution for the DOS of the whole system. Besides, the deformations for the DOS between the isolated CO molecule and the adsorbed one confirm that the CO molecule is activated during gas adsorption. Moreover, the large overlaps between DOS of Rh 4d and C 2p orbitals manifest strong hybridization between Rh and C atoms, which would explain the strong binding force of the Rh–C bond that leads to the short atom-to-atom distance.

### 3.3 NO adsorption

**Fig. 5:**
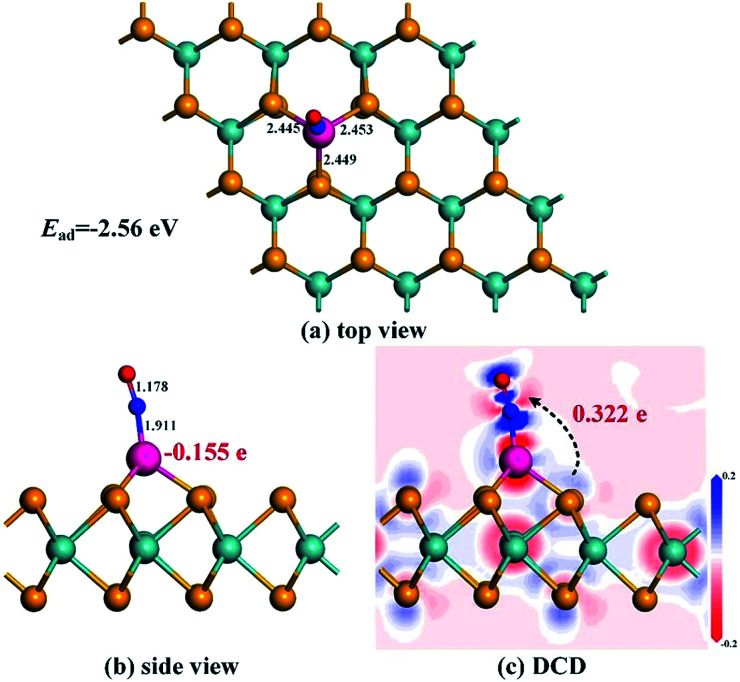
Adsorption configuration (a) and (b) and DCD (c) of the Rh-MoSe_2_/NO system.

The most stable adsorption configuration and related DCD for the Rh-MoSe_2_/NO system are exhibited in Fig. 5. One can observe that the structure for NO adsorption onto the Rh-MoSe_2_ monolayer, with N atoms captured, is somewhat similar to that of the CO system. However, the largely elongated Rh–Se bonds with lengths of 2.445, 2.453 and 2.449 Å in the NO system suggest a stronger binding force of Rh on the NO molecule than the CO molecule. Similarly, the elongated N–O bond of 1.178 Å in the adsorbed NO molecule in comparison with its isolated counterpart (1.164 Å) indicates the activation behavior of the NO molecule for interaction with the Rh-MoSe_2_ monolayer.^35^ The shorter length of the Rh–N bond (1.911 Å) compared with the sum of relevant covalent radii (1.96 Å for Rh–N^49^) shows the nature of chemisorption. In fact, all these results could be well supported by the large *E*_ad_ of −2.56 eV and *Q*_T_ of −0.322 e calculated in this system as they are large enough to confirm the strong interaction between gas molecules and the adsorbent surface.^51^

**Fig. 6:**
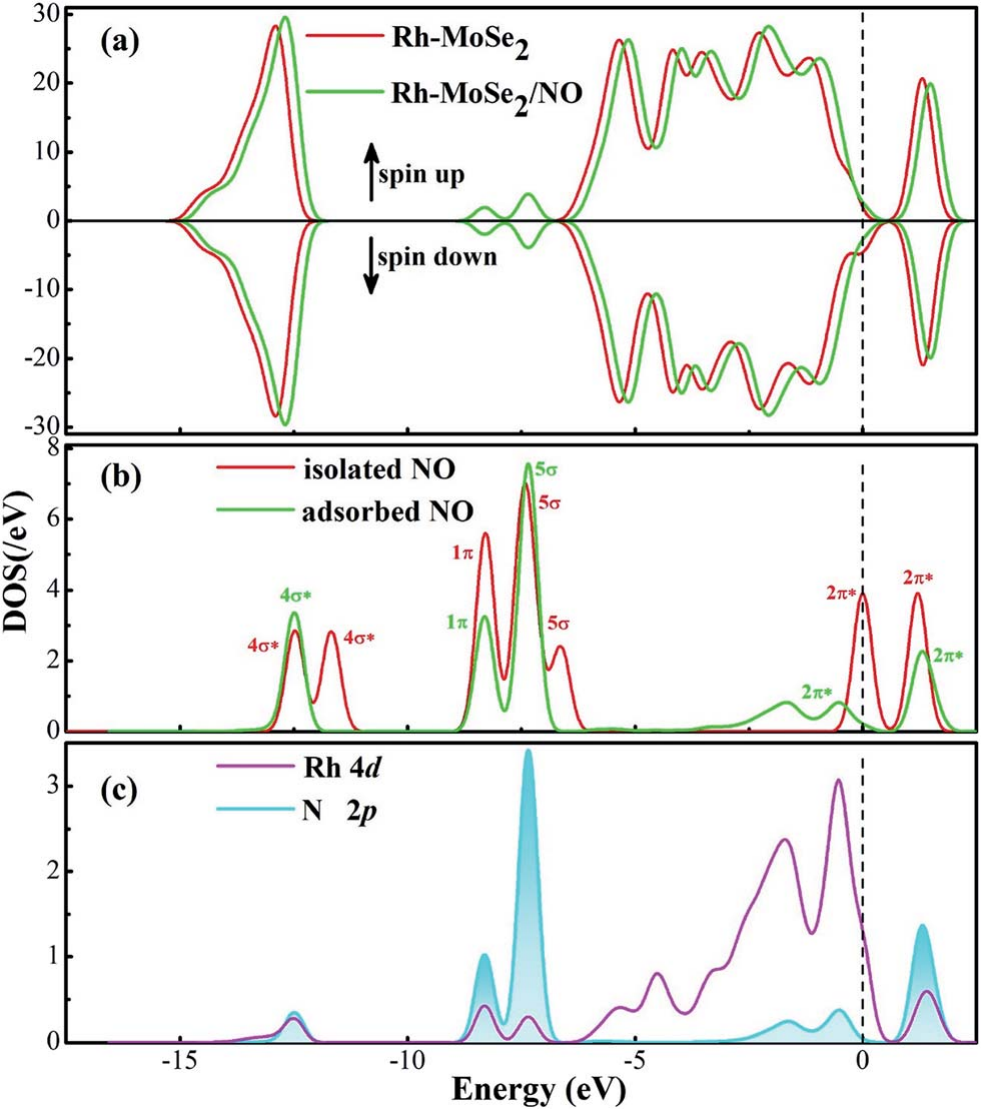
DOS distribution of the Rh-MoSe_2_/NO system. The dashed line is the Fermi level. In the gas DOS, the red value shows the orbitals of isolated molecules while the green one shows the orbitals of adsorbed molecules.

From the DOS distribution for the NO system as portrayed in Fig. 6, the electronic behavior for NO adsorption on the Rh-MoSe_2_ monolayer could be elucidated clearly. In the total DOS distribution, we can see that the DOS of the NO system transforms slightly above the Fermi level compared with that of the pure Rh-MoSe_2_ system. In detail, two novel peaks appear at around −7.5 and −8.5 eV while one peak at the Fermi level disappears after gas adsorption, which could be attributed to the DOS deformation of the adsorbed NO molecule. It could be seen that the DOS peak of the isolated NO molecule at the Fermi level splits into two small peaks after NO adsorption that weakens the contribution to the total DOS of the whole system, while the slightly weakened peak at −8.5 eV and the enhanced peak at −7.5 eV accord with the novel peaks in DOS for the adsorbed system. Based on the partial DOS analysis, we could see that every peak of the N 2p orbital is overlapped with that of the Rh 4d orbital, implying that the N atom is strongly hybridized with the Rh dopant, thus giving rise to a strong binding force for the Rh–N bond.

### 3.4 NO_2_ adsorption

**Fig. 7:**
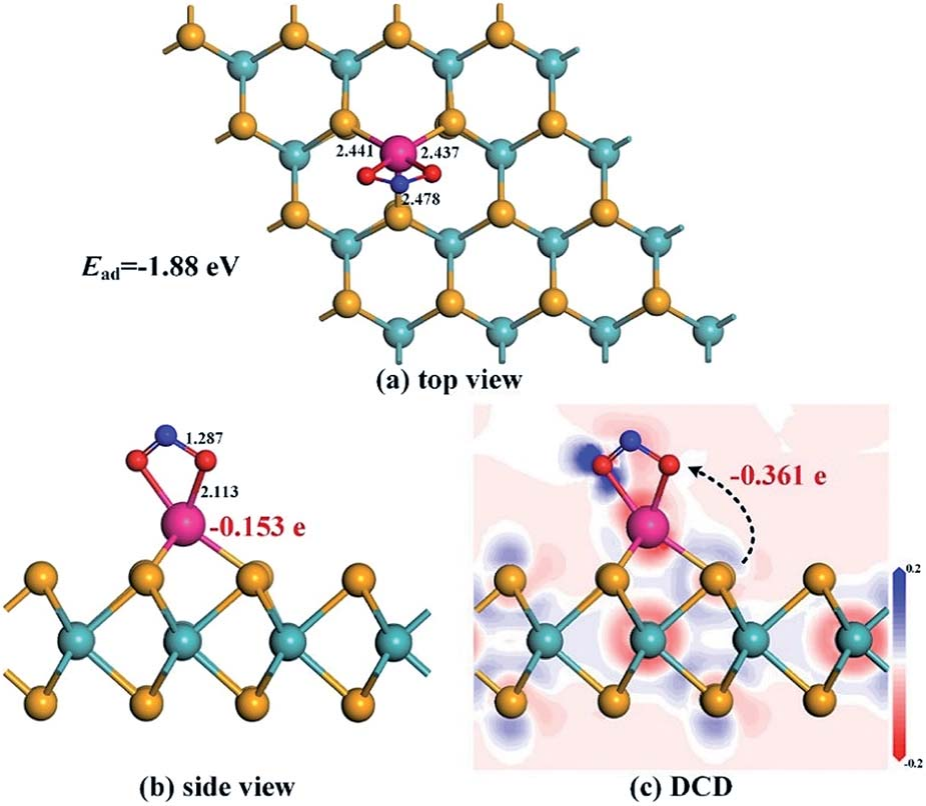
Adsorption configuration (a) and (b) and DCD (c) of the Rh-MoSe_2_/NO_2_ system.

In terms of NO_2_ adsorption onto the Rh-MoSe_2_ monolayer, one can see from Fig. 7 that the NO_2_ molecule prefers to adsorb two O atoms trapped with a shorter Rh–O distance of 2.113 Å. Three Rh–Se bonds of Rh-MoSe_2_ in line with the N–O bond of the NO_2_ molecule undergo some elongation after adsorption, due to the binding force of Rh on the NO_2_ molecule. The Mulliken population analysis indicates that 0.361 e transfers from the Rh-MoSe_2_ monolayer to the NO_2_ molecule with an electron loss of 0.086 for the Rh dopant. That is to say, unlike in the CO system, the Rh dopant acts as an electron donor releasing the electron to the gas molecule, which could be found in NO and SO_2_ systems as well. This finding manifests the strong electron mobility and chemical activity of TM when interacting with gas molecules.^52^ Moreover, the large *Q*_T_ associated with the relatively large *E*_ad_ suggests the kind of chemisorption for the Rh-MoSe_2_/NO_2_ system.

**Fig. 8:**
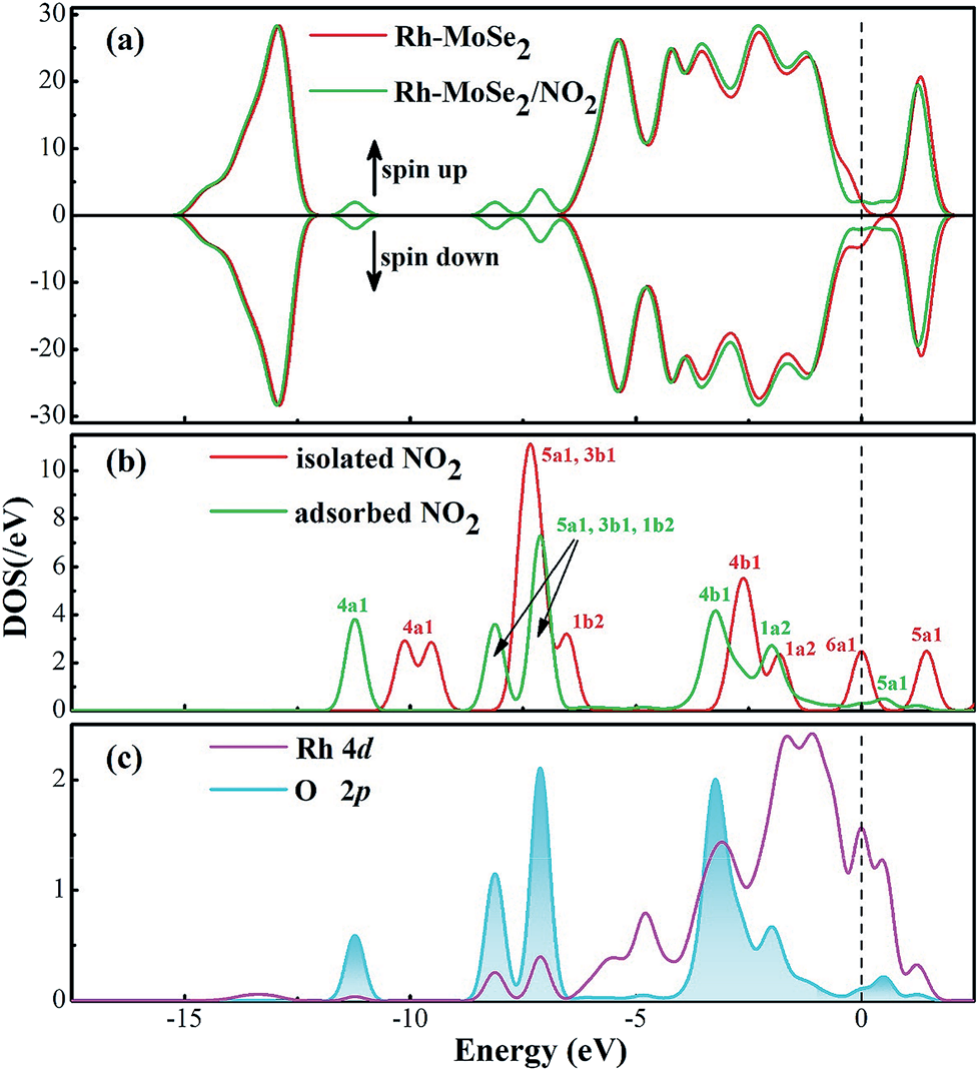
DOS distribution of the Rh-MoSe_2_/NO_2_ system. The dashed line is the Fermi level. In the gas DOS, the red value shows the orbitals of isolated molecules while the green one shows the orbitals of adsorbed molecules.

Fig. 8 shows the DOS distributions for the NO_2_ adsorption system. One can find from the total DOS configurations that the DOS curve transforms dramatically around the Fermi level after adsorption of the NO_2_ molecule. This may be attributed to the considerable electron-transfer between gas molecules and the adsorbent surface that results in electron redistribution for the whole system, thus leading to the deformation of electronic states at the Fermi level.^53^ Furthermore, the novel emerged peaks at −7.5, −8.5 and −11.5 eV are contributed by the adsorbed NO_2_ molecule which is activated during adsorption. Similarly, the hybridization between Rh and O atoms is identified through the phenomenon of overlaps between Rh 4d and O 2p orbitals.

### 3.5 SO_2_ adsorption

**Fig. 9:**
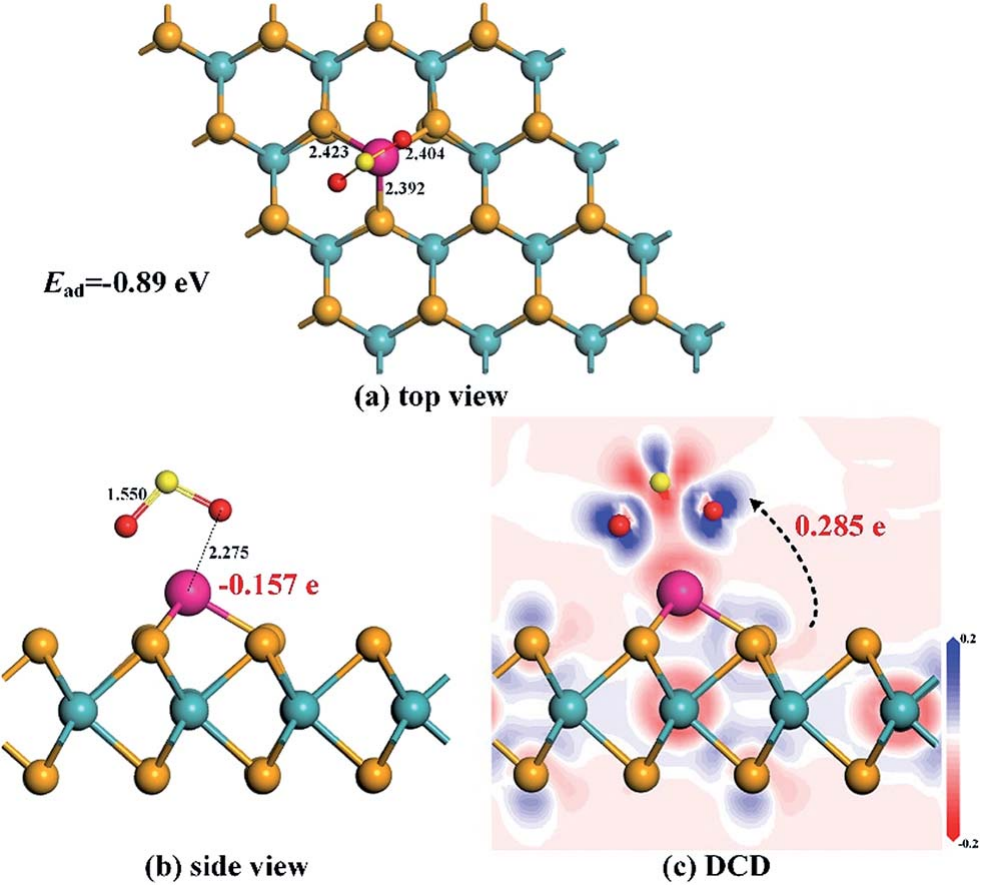
Adsorption configuration (a) and (b) and DCD (c) of the Rh-MoSe_2_/SO_2_ system.

From Fig. 9 where the most stable configuration for SO_2_ adsorption on the Rh-MoSe_2_ monolayer is displayed, we can see that, similar to the NO_2_ adsorption configuration, the SO_2_ molecule is adsorbed on the Rh-MoSe_2_ monolayer with two O atoms oriented. However, the SO_2_ molecule here stands a little far from the Rh dopant, with the nearest atom-to-atom distance measured to be 2.275 Å, which indicates a weak interaction in the Rh-MoSe_2_/SO_2_ system. The tiny prolongation for Rh–Se bonds inner Rh-MoSe_2_ to 2.423, 2.404 and 2.392 Å, along with S–O bond inner SO_2_ to 1.550 Å by 0.019 Å can confirm the weak binding force between the Rh dopant and the SO_2_ molecule as well. The *E*_ad_ obtained here is −0.89 eV and the Mulliken population analysis shows a *Q*_T_ of 0.285 e from the Rh-MoSe_2_ monolayer to the SO_2_ molecule. All these findings suggest physisorption for SO_2_ adsorption on the Rh-MoSe_2_ monolayer.^51^

**Fig. 10:**
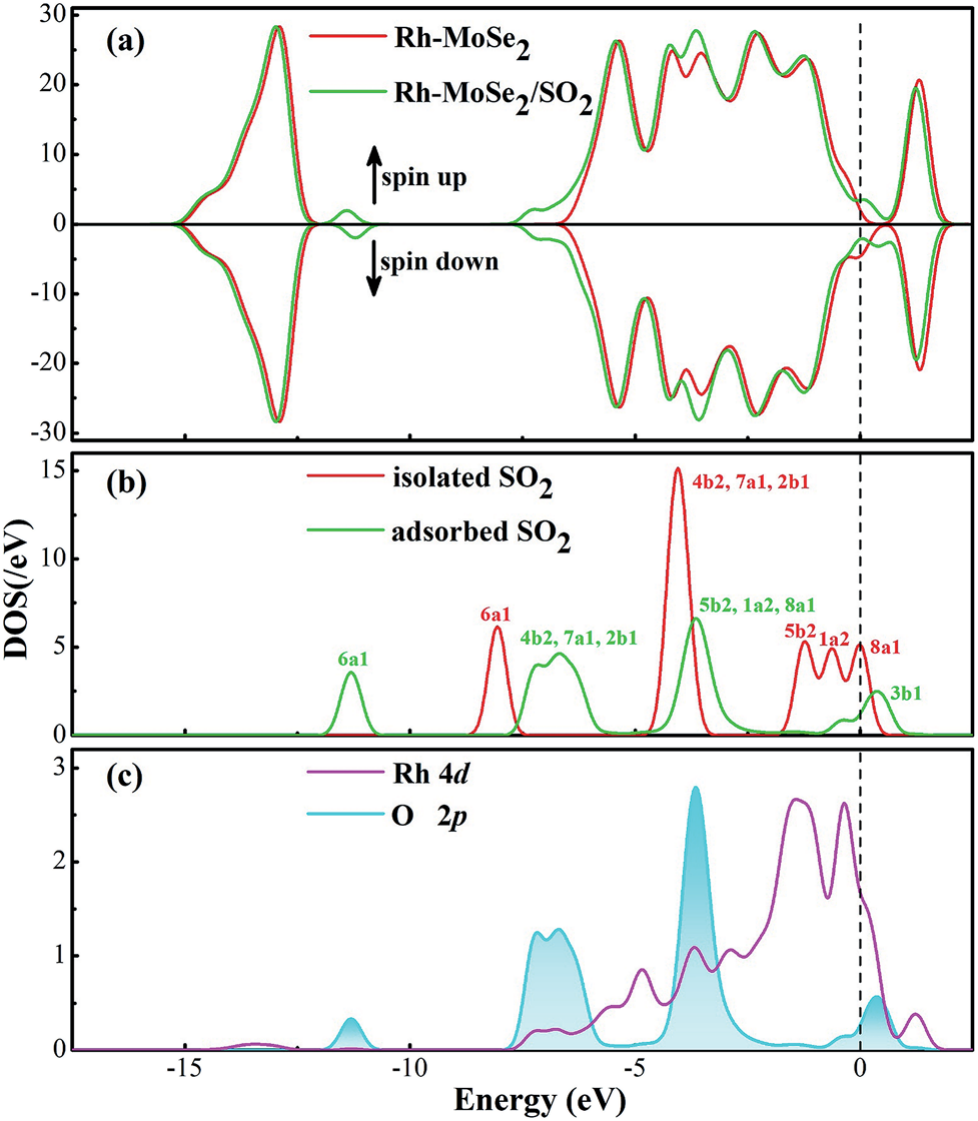
DOS distribution of the Rh-MoSe_2_/SO_2_ system. The dashed line is the Fermi level.

DOS distributions shown in Fig. 10 could give a clear explanation for the electronic behavior in this system. The novel peaks around −11.5 and −7 and the Fermi level in total DOS of the adsorbed system are from the contribution of the adsorbed SO_2_ molecule that is somewhat activated after adsorption. At the same time, the overlaps between DOS of Rh 4d and O 2p orbitals, although not as large as the NO_2_ system, prove the orbital interaction, to some extent, between the Rh dopant and the SO_2_ molecule.

### 3.6 Application of Rh-MoSe_2_ to toxic gas scavenging

We first investigated the potential application of Rh-MoSe_2_ as a resistance-type sensor for these gases. According to our previous analysis, it could be found that the Rh-MoSe_2_ monolayer has quite strong adsorption performance for three species namely CO, NO and NO_2_ molecules, giving rise to chemisorption in these systems, while having relatively weaker performance towards SO_2_ adsorption that gives rise to physisorption instead. In other words, it would be difficult for these three gas molecules to desorb from the Rh-MoSe_2_ monolayer once they are adsorbed onto the surface, except for the SO_2_ molecule which may be desorbed through annealing at high temperature or irradiation with ultraviolet light.^50,54^

**Fig. 11:**
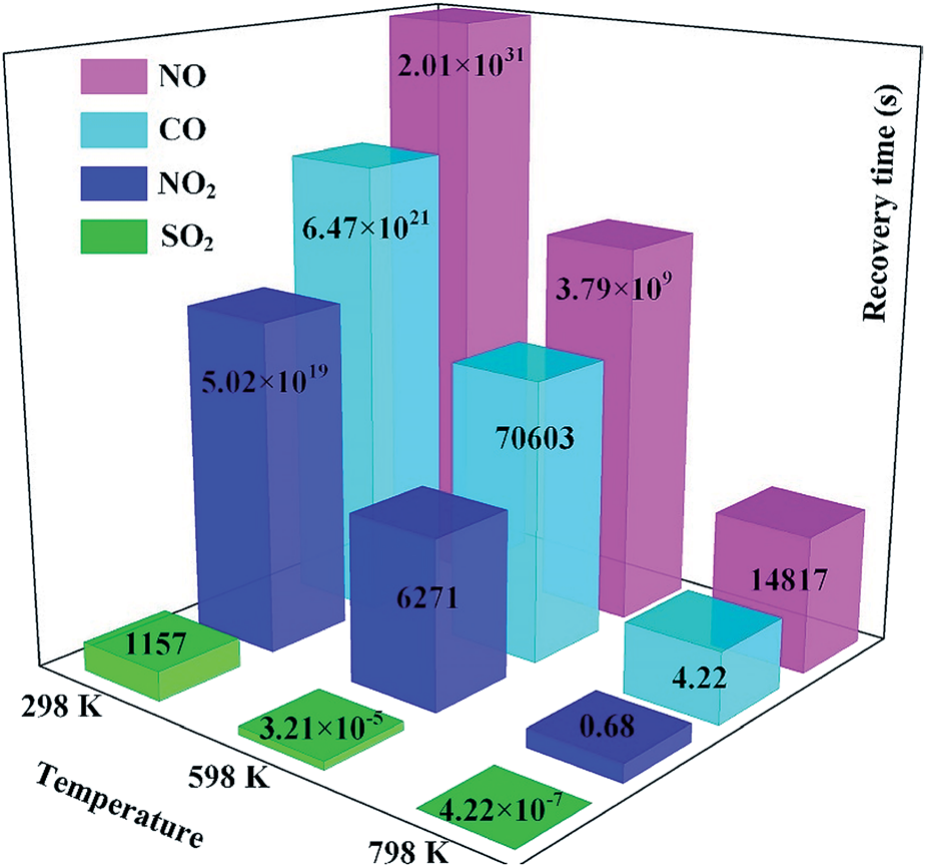
Recovery time for four species at various temperatures.

To confirm this assumption, the recovery time (*τ*) analysis based on transition state theory and van't-Hoff–Arrhenius expression^55^ was implemented, and expressed as:

3 *τ* = *A*^−1^e^(−*E*_a_/*K*_B_*T*)^

where *A* is the attempt frequency determined as 10^12^ s^−1^ according to a previous report,^56^*T* is the temperature and *K*_B_ is the Boltzmann constant (8.318 × 10^−3^ kJ (mol K)^−1^). Given the inverse processes between adsorption and desorption, we assume the value of *E*_ad_ as the potential barrier (*E*_a_) for desorption. It would be obvious that a larger *E*_ad_ would lead to a harder process for gas desorption, and the increase of temperature can accelerate that process effectively. According to the obtained *E*_ad_ in our calculations as shown in Table 1, we plotted the recovery time for the desorption of various gases at various temperatures as portrayed in Fig. 11. One can conclude that CO, NO and NO_2_ desorption from the Rh-MoSe_2_ monolayer would be extremely unrealistic at room temperature, and even at 798 K, it would take more than 4 hours for NO desorption from the surface. Although remarkably enhanced behavior for CO and NO_2_ desorption could be achieved at 798 K, the considerable heat loss and the safety of the devices would be another issue. Therefore, it would be inappropriate to use the Rh-MoSe_2_ monolayer as the sensing material for CO, NO or NO_2_ detection, because the one-off operation for gas sensors would be a waste of money and reduce work-efficiency. On the other hand, we assume that Rh-MoSe_2_ is suitable for SO_2_ sensing given its good adsorption performance for detection and desorption performance with appropriate recovery time at ambient temperature for recycle use. Moreover, the sensing mechanism of the Rh-MoSe_2_ monolayer towards SO_2_ would be dependent on the increased conductivity due to the narrowed energy gap from 1.033 eV for the isolated Rh-MoSe_2_ monolayer to 0.227 eV for the SO_2_ system^57^ as seen in Table 1.

**Table 1:** Adsorption parameters for various Rh-MoSe_2_/gas systems

Gas systems	*E*_ad_ (eV)	*D* (Å)	*Q*_T_ (e)	*E*_g_ (eV)
CO	−2.00	1.892	−0.131	0.202
NO	−2.56	1.911	0.322	1.210
NO_2_	−1.88	2.113	−0.361	0.520
SO_2_	−0.89	2.275	0.285	0.227

**Fig. 12:**
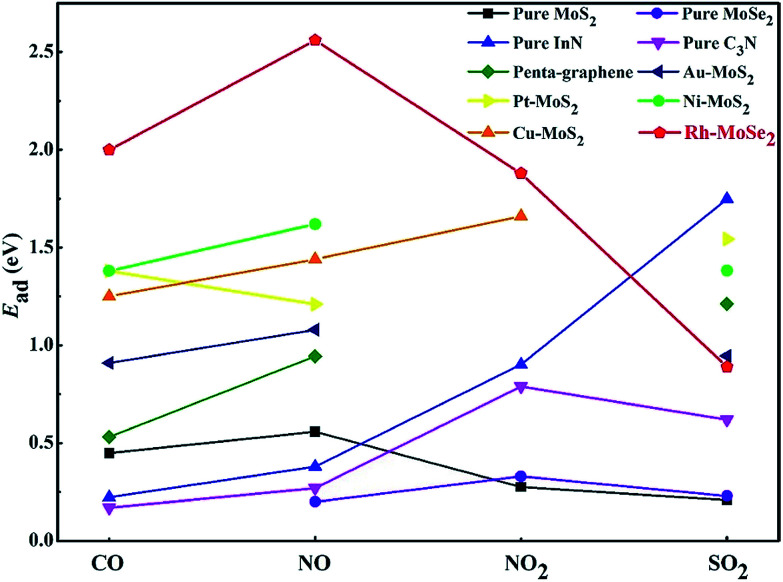
*E*_ad_ comparison of Rh-MoSe_2_ for four species with recent reports.

Second, the large *E*_ad_ in CO, NO and NO_2_ systems allows the excellent performance of Rh-MoSe_2_ as a gas adsorbent for their storage or removal from specific environments. For this purpose, we summarize in Fig. 12 the calculated *E*_ad_ values of recently reported 2D adsorbents for four gases, in order to find out whether Rh-MoSe_2_ could be an alternative for scavenging toxic gases. From the figure, we can find that the Rh-MoSe_2_ monolayer possesses better adsorption performance for CO, NO and NO_2_ molecules compared with the pure MoS_2_ monolayer,^23^ the pure MoSe_2_ monolayer,^58^ pure InN,^19^ pure C_3_N,^57^ pure penta-graphene,^59^ and some other TM-doped MoS_2_ monolayers.^22,40,60,61^ That is to say, the Rh-MoSe_2_ monolayer has strong potential to be an adsorbent candidate for CO, NO or NO_2_ storage and removal. Conversely, it is not a desirable SO_2_ adsorbent due to its weaker performance than pure InN,^19^ penta-graphene,^59^ the Au-MoS_2_ monolayer,^40^ the Pt-MoS_2_ monolayer^40^ and the Ni-MoS_2_ monolayer,^60^ although it has better performance than the pure MoS_2_ monolayer,^23^ the pure MoSe_2_ monolayer^58^ and pure C_3_N,^57^ except for the Cu-MoS_2_ monolayer which is not studied for SO_2_ adsorption in ref. 22 and 61. In addition, we would introduce the application of borophene as a potential SO_2_ adsorbent given its strong ability and capacity whereby up to seven SO_2_ molecules could be chemisorbed on its one side, with a weight percentage of 82.88%, for SO_2_ adsorption.^62^

## 4. Conclusions

In this work, we implemented a first-principles theory to study the adsorption performance of the Rh-MoSe_2_ monolayer towards four toxic gases, including CO, NO, NO_2_ and SO_2_. Desorption behavior analysis and adsorption behavior comparison with other 2D materials towards these four species were conducted in order to exploit the potential application of our proposed monolayer. The results indicated that the Rh-MoSe_2_ monolayer possesses quite strong adsorption behavior towards CO, NO and NO_2_ molecules that gives rise to chemisorption in these systems, while physisorption could be determined for the Rh-MoSe_2_/SO_2_ system. In that regard, based on the adsorption behavior comparison with other adsorbents and desorption behavior analysis, we assume that Rh-MoSe_2_ is a desirable adsorbent for CO, NO and NO_2_ storage or removal while it is a good sensing material for SO_2_ detection. Our theoretical calculation would provide a first insight into the TM-doping effect on the structural and electronic properties of the MoSe_2_ monolayer, and shed light on the application of Rh-MoSe_2_ for the sensing or disposal of common toxic gases.

## Author contributions

Xiaoxing Zhang conceived and designed the research, Hao Cui performed the research and wrote this manuscript while Guozhi Zhang and Ju Tang helped analyze the data.

## Conflicts of interest

There are no conflicts to declare.

## Supplementary Material
